# Tumor Microenvironment-Responsive Shell/Core Composite Nanoparticles for Enhanced Stability and Antitumor Efficiency Based on a pH-Triggered Charge-Reversal Mechanism

**DOI:** 10.3390/pharmaceutics13060895

**Published:** 2021-06-16

**Authors:** Qiuhua Luo, Wen Shi, Puxiu Wang, Yu Zhang, Jia Meng, Ling Zhang

**Affiliations:** 1 Department of Pharmacy, The First Affiliated Hospital of China Medical University, Shenyang 110001, China; qhluo@cmu.edu.cn (Q.L.); 2020121055@stu.cmu.edu.cn (W.S.); pxwang@cmu.edu.cn (P.W.); 2 School of Pharmacy, China Medical University, Shenyang 110122, China; 3 Department of Pharmaceutics, College of Pharmacy, Shenyang Pharmaceutical University, Shenyang 110016, China; zhangyu@syphu.edu.cn; 4 Department of Pharmacy, Liaoning Institute of Basic Medicine, Shenyang 110101, China; nancy-1201@163.com; 5 Department of Biotherapy, Cancer Research Institute, The First Affiliated Hospital of China Medical University, Shenyang 110001, China

**Keywords:** shielding system, shell/core structure, charge-reversal, pH-triggered, cancer therapy

## Abstract

High systemic stability and effective tumor accumulation of chemotherapeutic agents are indispensable elements that determine their antitumor efficacy. PEGylation of nanoparticles (NPs) could prolong the retention time in vivo by improving their stability in circulation, but treatment suffers reduced tumor penetration and cellular uptake of nanomedicines. The tumor microenvironment (TME)-responsive NPs maintain their stealth features during circulation and undergo a stimuli-responsive dePEGylation once exposed to the site of action, thereby achieving enhanced internalization in tumor cells. Herein, TME-responsive shell/core composite nanoparticles were prepared and optimized with enhanced stability and tumor intake efficiency. We synthesized 12-hydroxystearic acid-poly (ethylene glycol)-YGRKKRRQRRR (HA-PEG-TAT) as a post-insert apparatus in disulfiram (DSF)-encapsulated naked nanoparticles (N-NPs) in order to form a cationic core (TAT-NPs). Accordingly, the negatively charged poly (glutamate acid)-graft-poly (ethylene glycol) (PGlu-PEG) was further applied to the surface of TAT-NPs as a negative charged shell (PGlu-PEG/TAT-NPs) via the electrostatic interaction between glutamic acids and arginine at the outer ring of the TAT-NPs. PGlu-PEG/TAT-NPs displayed a huge loading capability for DSF with reduced degradation in plasma and exhibited rapid charge reversal when pH decreased from 7.4 to pH 6.5, demonstrating an excellent systemic stability as well as intelligent stimuli-responsive performance within the acidic TME. Furthermore, the in vivo antitumor study revealed that PGlu-PEG/TAT-NPs provided greater antitumor efficacy compared with free DSF and N-NPs with no obvious systemic toxicity. In conclusion, the TME-responsive shell/core composite NPs, consisting of PGlu-PEG and HS-PEG-TAT, could mediate an effective and biocompatible delivery of chemotherapeutic agents with clinical potential.

## 1. Introduction

Coating the surface of nanoscale drug delivery systems (NDDS) with polyethylene glycol (PEG)—namely, PEGylation—has been extensively applied to inhibit protein adsorption and prevent rapid elimination [1]. The PEGylation strategy thus improves pharmacokinetics of NDDS with prolonged circulation, an enhanced permeability and retention (EPR) effect, and reduced premature drug leakage, while also decreasing collateral damage to normal healthy tissues [2,3]. However, PEGylation hinders the internalization of tumors, resulting in limited tumor accumulation, which is referred to as the “PEG dilemma” [4]. Therefore, it is of utmost importance to develop NDDS with the ability of PEGylation and dePEGylation according to the requirements.

The tumor microenvironment (TME) presents several distinguishable pathological features such as mildly acidic pH, hypoxia, high level of reactive oxygen species, and overexpression of specific enzymes, which have been exploited as stimuli to induce dePEGylation [5,6,7]. To sum up, PEGylation/dePEGylation strategies involve chemical and physical approaches. Chemical approaches mainly refer to conjugating ligands and PEG via cleavable bonds (such as acid labile chemical bond, disulfide or short peptide containing enzyme-consensus) that respond to the TME [8,9,10]. Physical approaches could be summarized as charge a conversion strategy, which relies on non-covalent adsorption of PEG to the nanoparticle surface [11,12]. In this way, the use of anionic natural polymers with charge properties, including polyglutamic acid and hyaluronic acid, gives assistance to the construction of a charge-reversal system [13,14]. Polyglutamic acid-based zwitterionic copolymers own abundant carboxylic moieties of glutamic chains to be interacted with cationic moieties [15]. Once exposed to an acidic environment, the carboxylic groups are protonated immediately, leading to the disruption of the above electrostatic interactions; simultaneously, the coil-like conformation of polyglutamic acid is transformed into an α-helix one [16]. Therefore, by utilizing charge properties of deprotonation/protonation, polyglutamic acid-based zwitterionic copolymers could be applied in stimuli-responsive nanomedicine through electrostatic attraction for achieving the conversion of PEGylation/dePEGylation.

As for ligand-modified active-targeted delivery systems, cell-penetrating peptides (CPPs) with the distinct advantages of positive charges have been used to ferry molecules or nanomedicines into cells independent of classical endocytosis, while they suffer from non-specificity that may produce toxic side effects in normal tissues [17,18]. Therefore, it is of great significance to introduce a dePEGylation strategy into a CPPs-NDDS, aiming to take advantage of the penetrating advantage of CPPs confined to the tumor site and to avoid toxicity effects at normal sites.

Disulfiram (DSF) is a potential anticancer drug, but it is limited by its poor plasma stability in circulation [19,20]. So far, several DSF-loaded NDDS have been developed for enhanced therapeutic effects [21,22]. Zhuo et al. fabricated an injectable nanoparticle composed of mPEG_5000_-PCL_5000_ and MCT that increased DSF loading from 3.35% to 5.5% with a more stable release profile, which effectively inhibited H22 xenograft tumor growth in vivo [23]. Similarly, Miao et al. developed DSF-incorporated mPEG_5k_-b-PLGA_2k_/PCL_3.4k_/MCT micelles that increased the tumor inhibition rate of DSF by nearly 3.6 times compared with that of the DSF solution, primarily benefiting from a prolonged half-life of DSF [24]. In addition, DSF nanosuspensions with soybean lecithin (DSF/SPC-NSps) exhibited high drug capacity (44.39 ± 1.09%) and prominent stability in physiological media that avoided the degradation and clearance from the RES [25]. Moreover, folate-receptor-targeted PLGA-PEG nanoparticles also indicated excellent encapsulation efficiency of DSF (59.62%) and enhanced tumor targeting ability, compared with naked NPs and DSF solution [26].

**Scheme 1:**
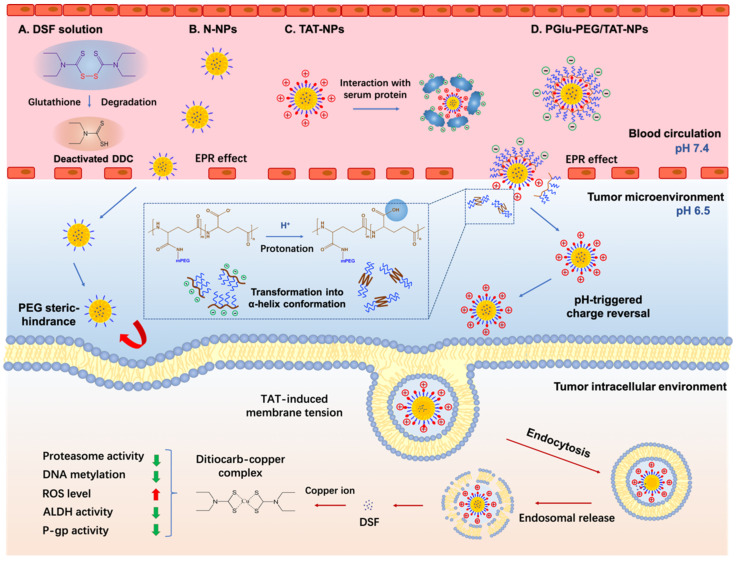
Schematic illustration of tumor microenvironment-responsive DSF-loaded nanoparticles for enhanced stability and antitumor efficiency based on a pH-triggered charge-reversal mechanism. DSF in solution or TAT-NPs could interact with glutathione or serum protein, leading to short half-life in blood circulation. The PEG steric hindrance on N-NPs could limit the intracellular uptake of DSF. PGlu-PEG/TAT-NPs could target to tumor sites and dePEGylate to TAT-NPs due to the protonation of PGlu-PEG to accomplish pH-triggered charge reversal in the tumor microenvironment; TAT-induced cellular uptake increases the accumulation of intracellular DSF, which could form ditiocarb–copper complex to inhibit tumor progression through multiple pathways. N-NPs: DSF-loaded naked nanoparticles, composed of DSF, medium-chain triglycerides, macrogol 15 hydroxystearate and lecithin; TAT-NPs: DSF-loaded cationic nanoparticles, composed of HS-PEG-TAT and N-NPs; PGlu-PEG/TAT-NPs: DSF-loaded shell/core composite nanoparticles, composed of PGlu-PEG and TAT-NPs. DDC, diethyldithiocarbamic acid; ROS, reactive oxygen species; ALDH, aldehyde dehydrogenase; P-gp, P-glycoprotein.

In order to better play the strengths of DSF, we explored a DSF delivery system in the form of shell/core composite NPs and endowed it with a pH-triggered charge-reversal property. TAT, a widely used CPP, was conjugated to 12-hydroxystearic-poly (ethylene glycol) (HS-PEG-TAT) and was applied to modify DSF-loaded naked nanoparticles (N-NPs) by the post-insertion method to obtain cationic nanoparticles (TAT-NPs). PGlu-PEG was applied to form a corona shell via the electronic interaction with the moieties of TAT, aiming to protect TAT and inner DSF from serum protein absorption (Scheme 1). The shell/core composite nanoparticles (PGlu-PEG/TAT-NPs) were characterized in terms of particle size, zeta potential, drug loading, entrapment efficiency, and particle morphology. PGlu-PEG/TAT-NPs displayed a huge loading capability for DSF with reduced degradation in plasma and exhibited rapid charge reversal when pH decreased from the physiological condition to the TME. Cell uptake study indicated that the pH responsiveness of PGlu-PEG/TAT-NPs contributed to the exposure of TAT and facilitated the internalization of NPs in tumor cells. The processing conditions and composition ratios for TAT peptide and PGlu-PEG were optimized to achieve an improved antitumor effect of DSF in vivo. This investigation laid a foundation for developing an intelligent DSF delivery system by making use of a shell/core structure with the advantages of dePEGylation provided by the modification of TAT and PGlu-PEG.

## 2. Materials and Methods

### 2.1. Materials

DSF was purchased from Jinan Ruijing Pharmaceutical Co. Ltd. (Jinan, China). Medium-chain triglycerides were purchased from Tieling Pharmaceutical Co. Ltd. (Tieling China). Macrogol 15 hydroxystearate (Kolliphor HS15) was provided by BASF AG (Ludwigshafen, Germany). 12-hydroxystearic, NHS-PEG16-maleimide, and TAT (AYGRKKRRQRRR) was obtained from ChinaPeptides Co. Ltd. (Shanghai, China). Poly (glutamate acid)-graft-poly (ethylene glycol) (PGlu-PEG, MW: PGlu 10,000 Da, PEG 2000 Da, graft ratio 8%) was provided by Changchun Institute of Applied Chemistry (Changchun, China). Cy5.5 was purchased from Seebio Biotech Co. Ltd. (Shanghai, China). Dulbecco’s modified Eagle’s medium (DMEM), RPMI-1640, and fetal bovine serum (FBS) were purchased from Gibco (Grand Island, NY, USA). MTT (3-(4,5-dimethylthiazol-2-yl)-2,5-diphenyl tetrazolium bromide) and copper gluconate were purchased from Sigma-Aldrich (St. Louis, MO, USA). All other reagents were of analytical grade.

### 2.2. Cell Lines and Cell Cultures

The human hepatocellular carcinoma cell line Hep G2, human breast cancer cell line MCF7, fibroblasts (L929), and murine hepatoma 22 (H22) cells were obtained from the Chinese Academy of Sciences cell bank (Shanghai, China). Hep G2 and MCF7 cells were cultured in DMEM supplemented with 10% FBS and 3 μM copper gluconate. L929 cells were cultured in RPMI-1640 medium with 10% FBS. Cells were cultured at 37 °C in a humidified 5% CO_2_ incubator. All cells were used for study at 3–7 passages.

### 2.3. Preparation and Characteristics of DSF-Loaded Nanoparticles

DSF-loaded naked nanoparticles (N-NPs) were prepared by the phase-inversion method. Briefly, the mixture of DSF, medium-chain triglycerides, macrogol 15 hydroxystearate, lecithin, NaCl, and water was heated to complete solubilization at 60 °C and then subjected to three heating cycles between 60 °C and 80 °C, followed by adding deionized water (0 °C) 3 times of volume into the system. Then, the system was transferred to an ice-water bath for agitation to the formation of N-NPs. TAT-modified cationic nanoparticles (TAT-NPs) were prepared by repetitively incubating the mixture of HS-PEG-TAT and N-NPs in hot water bath and quenching in an ice bath. The temperature of the hot water bath and the period of incubation were optimized according to the changes of particle size and zeta potential. The free HS-PEG-TAT was removed by Sephedex G50 column chromatography. TAT-NPs with different TAT concentrations (0.1, 0.2, 0.6, 1.2 and 2%, *w*/*w*) were prepared by adding a series percentage of HS-PEG-TAT as in the method above.

Shell/core composite nanoparticles (PGlu-PEG/TAT-NPs) were prepared by adding TAT-NPs into PGlu-PEG solution (adjusted to pH 8–9) dropwise with stirring and were kept stirred for another 4 h. Then, free PGlu-PEG was removed by dialysis. PGlu-PEG/TAT-NPs with different PGlu-PEG percentages (2, 5, and 10%, *w*/*w*) were prepared by adding a series concentration of PGlu-PEG solution to TAT-NPs as in the method above.

The particle size and zeta potential of nanoparticles with different modifications of TAT and PGlu-PEG were measured by Nicomp 380 ζ potential and a particle sizer (Nicomp Particle Sizing Systems, Santa Barbara, CA, USA). The morphology of different nanoparticles was observed by using a JEOLJEM-2000EX transmission electron microscope equipped with an energy-dispersive spectroscopy (EDS) Si (Li) detector.

The content of DSF was determined by the HPLC-UV method. The chromatographic separation was carried out on a C18 column (4.6 × 200 mm, 5 μm, Dikama Technologies, Beijing, China). The temperature of the column, detection wavelength, and injection volume were 25 °C, 254 nm, and 20 μL, respectively. The mobile phase consisted of methanol and water at a ratio of 70:30. The drug loading (DL, %) and entrapment efficiency (EE, %) were calculated as follows:

DL (%) = (weight of DSF/(weight of DSF + weight of lipid materials)) × 100%

EE (%) = (1 − weight of untrapped DSF/weight of total DSF) × 100%

### 2.4. pH-Responsive Charge Conversion Study

N-NPs, TAT-NPs, and PGlu-PEG/TAT-NPs were placed in a dialysis bag (14,000 MWCO, Greenbird Technology Development Co. Ltd, Shanghai, China) and dialyzed separately against PBS at pH 7.4 and 6.5. At the indicated time, solution was withdrawn from the incubation medium for the determination of zeta potential.

### 2.5. Plasma Stability Study

The colloidal stability of DSF loaded N-NPs, TAT-NPs, and PGlu-PEG/TAT-NPs in plasma was evaluated through a transmittance study as described previously [27]. Briefly, 0.5 mL of DSF-loaded formulations were diluted with 2 mL PBS containing 50% rat plasma, respectively. Rat plasma was collected from the whole blood of Sprague-Dawley (SD) rats. The whole blood was treated with sodium heparin, then vortexed for 30 s and centrifuged at 6000 rpm for 10 min to extract the supernate as rat plasma. PGlu-PEG/TAT-NPs was further divided to two groups separately against PBS at pH 7.4 and 6.5 containing 50% rat plasma. The transmittance of the formulations was determined at 660 nm at predetermined intervals.

Additionally, the stability of DSF in different nanoparticles evaluated by the residual content of DSF. Briefly, 0.5 mL of DSF-loaded formulations were added into rat plasma (2 mL) in tubes at 37 °C with a shaking rate of 100 rpm. At predetermined intervals, aliquots of 200 μL were withdrawn, treated with 200 μL of acetonitrile, and vortexed for 1 min. The mixture solution was centrifuged at 12,000 rpm for 10 min at 4 °C. The supernatant was collected and analyzed to determine the DSF. DSF solution was prepared by dissolving DSF in Cremophor EL/ethanol and was then diluted with saline.

### 2.6. pH-Responsive Intracellular Uptake Study

Cy5.5 dye was used as a fluorescent indicator (replacing DSF) for visual observation to investigate the intracellular uptake of pH-responsive nanoparticles. The preparation of Cy5.5-labeled NPs (20 ng/mL) was consistent with that of DSF-loaded NPs, according to the preparation methods in Section 2.3, in order to obtain Cy5.5-labeled N-NPs, Cy5.5-labeled TAT-NPs, and Cy5.5-labeled PGlu-PEG/TAT-NPs for further fluorescent investigation. Adherent Hep G2 cells were incubated in 96-well culture plates at a density of 5 × 10^3^ cells/well overnight. Cy5.5-loaded nanoparticles were pretreated to corresponding pH values (pH 7.4 and 6.5) for 4 h. The medium was replaced by fresh medium containing different Cy5.5-loaded nanoparticles for 4 h. Then the cells were washed with cold PBS for three times and fixed with 4% paraformaldehyde solution. 4′,6′-diamidino-2-phenylindole (DAPI) was used to stain the cell nuclei for 20 min, followed by wash with PBS. Finally, the cells were photographed by an ImageXpressMicro high content system (Molecular Devices, San Jose, CA, USA). The images were quantified and analyzed using MetaXpress software (Molecular Devices, San Jose, CA, USA). The internalized Cy5.5 was measured with excitation/emission wavelengths set at 670 nm/700 nm.

### 2.7. Cytotoxicity Study

The cytotoxicity of DSF and DSF-loaded nanoparticles was studied by MTT assay as described previously [28]. In brief, Hep G2 and MCF7 cells were seeded in 96-well plates at a seeding density of 5 × 10^3^ cells/well overnight. PGlu-PEG/TAT-NPs were pretreated against PBS at pH 7.4 and pH 6.5 by PBS, respectively. After that, cells were constantly exposed to a series of concentrations of DSF and DSF-loaded nanoparticles for 24 h. Then, 10 μL MTT solution (5 mg/mL) was added to each well and incubated for another 4 h at 37 °C. The medium was completely removed, followed by adding DMSO (100 μL) to dissolve the formazan generated by the living cells. The cells without treatment were used as control. The absorbance of each well was measured on a microplate reader at a wavelength of 570 nm. The cell viability was calculated according to the formula: Cell viability (%) = (A_test_/A_control_) × 100%, where A_test_ and A_control_ were the absorbances of the test groups and control group.

### 2.8. In Vivo Antitumor Efficiency and Toxicity

Kunming mice (SPF grade, male, 18–22 g) were supplied from Shenyang Pharmaceutical University. All animal studies were carried out under Institutional Animal Care and Use Committee-approved protocols of Shenyang Pharmaceutical University (SYPU-IACUC-C2019-11-29-101). H22 tumor cells were intraperitoneally injected into healthy Kunming mice (0.2 mL/mouse), and the inoculation of ascites in the mice was observed. After the abdominal cavities were filled with ascites, mice were sacrificed, and the ascites were diluted 50 times with saline. The H22 xenograft tumor model was established by injecting the diluted ascites into the armpit region after disinfection.

When the tumor volume reached 50–80 mm^3^, the mice were randomly divided into four groups (*n* = 5) as follows: (1) saline (control), (2) DSF solution, (3) DSF-loaded N-NPs, (4) DSF-loaded PGlu-PEG/TAT-NPs. Saline (stroke-physiological saline solution) was applied as negative control. DSF solution was prepared by dissolving DSF in Cremophor EL/ethanol and then diluted with saline. At day 1, 3, 5, 7, 9, and 12, all groups of mice obtained intravenous injection of corresponding DSF injections at the dose of 20 mg/kg. Tumor volume (TV = length × width^2^/2) and body weight of mice were recorded every two days. After 21 days, mice were sacrificed to obtain the tumors and major organs (heart, liver, spleen, lung, and kidney) for pathological study. The tumors and organs were accurately weighted to calculate the tumor inhibition rate (TIR) by the following formula: TIR (%) = (1 − W_test_/W_control_) × 100. Then, tumor and organ sections were stained by hematoxylin and eosin (H&E) and were observed using an optical microscope observation.

### 2.9. Statistical Analysis

The statistical analysis was performed with Student’s *t*-test to compare differences between two treatment groups. The values of *p* < 0.05 was considered to be statistically significant.

## 3. Results and Discussion

### 3.1. Preparation and Characterization of DSF-Loaded Nanoparticles

**Figure 1:**
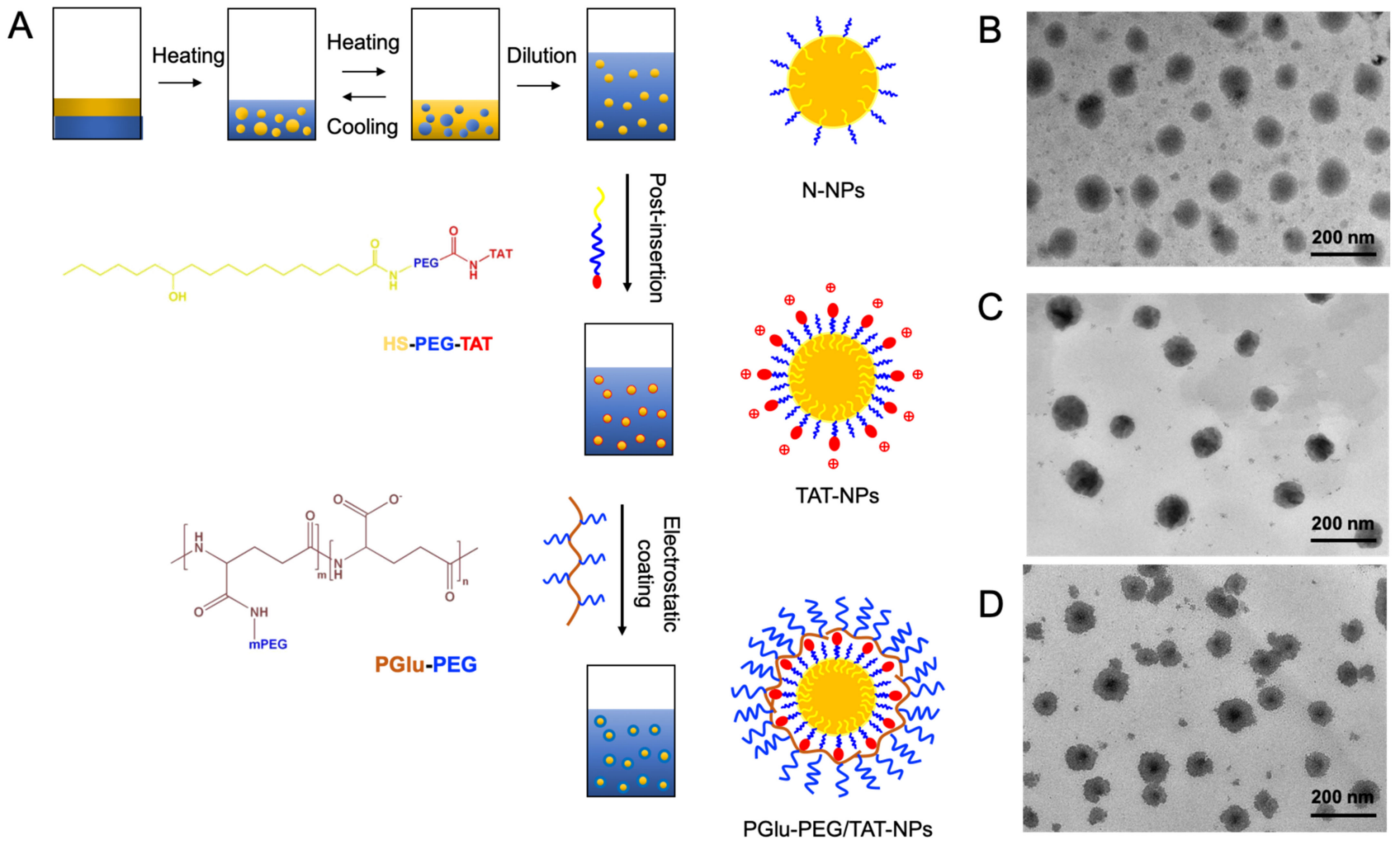
(**A**) Schematic illustration of preparation of DSF-loaded nanoparticles and corresponding structures. N-NPs were prepared by phase-inversion method. TAT-NPs were obtained by post-insertion of HS-PEG-TAT due to its lipophilicity of stearate. PGlu-PEG/TAT-NPs were manipulated by electrostatic absorption between -NH_3_^+^ and -COO^−^. (**B**–**D**) TEM images of DSF-loaded N-NPs (**B**), TAT-NPs (**C**), and PGlu-PEG/TAT-NPs (**D**). Scale bars: N-NPs and TAT-NPs: 200 nm; PGlu-PEG/TAT-NPs: 500 nm. HS-PEG-TAT, 12-hydroxystearic-PEG16-TAT AYGRKKRRQRRR; PGlu-PEG, poly (glutamate acid)-graft-poly (ethylene glycol); N-NPs, DSF-loaded naked nanoparticles; TAT-NPs, DSF-loaded cationic nanoparticles; PGlu-PEG/TAT-NPs, DSF-loaded shell/core composite nanoparticles.

DSF-loaded nanoparticles were prepared by the methods consisting of phase-inversion, post-insertion, and polyion complex (Figure 1A). The formulations of nanoparticles (F1 to F6) are displayed in Table 1. Specifically, F1 means N-NPs that were without TAT or PGlu-PEG modifications; F2 and F3 were TAT-NPs that were modified with different TAT concentrations (0.6% for F2 and 1.2% for F3) but without PGlu-PEG modification; F4, F5, and F6 were PGlu-PEG/TAT-NPs that were modified with the same TAT concentration (1.2%) and different PGlu-PEG concentrations (2% for F4, 5% for F5, and 10% for F6). Furthermore, we explored the effect of modification amount of TAT and PGlu-PEG on the particle size, zeta potential, drug loading, and entrapment efficiency to obtain the best formulation composition. As shown in Table 1, the increase of TAT content had an influence on the change of particle size (from 58.5 ± 1.2 nm to 65.4 ± 2.7 nm) and led to a harsh rise of zeta potential (from −16.43 mV to 6.69 mV). Since TAT is rich in arginine and lysine, the fact that zeta potential changed from negative to positive confirmed the successful insertion of the TAT peptide. When the concentration of TAT reached to 1.2%, the particle size and zeta potential remained basically unchanged. This phenomenon could be attributed to the saturated insertion sites for HS-PEG-TAT on the surface of nanoparticles. After electrostatic coating of PGlu-PEG, the particle size of shell/core composite nanoparticles increased ~22 nm compared with TAT-NPs (F6), and their zeta potential showed a conversion from positive values to negative values with a variation of 8–45 mV (F4–F6, Table 1), indicating the formation of a polyion complex coating layer. The drug loading (DL) and entrapment efficiency (EE) for DSF in nanoparticles of different formulations were above 4% and 90% (Table 1), respectively, suggesting a good loading capacity of DSF.

The morphology of DSF-loaded nanoparticles was determined by TEM. The DSF-loaded nanoparticles were uniform with sphere-shaped structures (Figure 1B–D). It is worth mentioning that PGlu-PEG/TAT-NPs showed an obvious shell/core structure, confirming the successful coating of PEG corona. Additionally, the particle sizes measured by TEM were slightly smaller than those from DLS (Figure 1D), which might be attributed to the dehydration of nanoparticles during the TEM sample preparation and shrinkage of the PEG corona.

Moreover, the incubation temperature has a decisive impact on the efficiency of post-insertion as reported before [29]. The insertion of TAT was shown to induce an increase of the particle size and zeta potential when the incubation temperature went up from 25 °C to 60 °C (Figure S2). The preparation process was further optimized through the regulation of temperature (25 °C, 37 °C, 45 °C, and 60 °C) and incubation time. The particle size and zeta potential indicated a positive correlation with the increase of incubation temperature and time, and both gradually reached to the maximum and maintained a steady value. To sum up, an incubation time of at least 120 min and a temperature of 60 °C were found to be necessary for the insertion of HS-PEG-TAT into nanoparticles.

As shown in Figure S3, the increase of TAT content had a slight influence on the change of particle size but led to a harsh rise of zeta potential (from −16.43 mV to 6.69 mV). Since TAT is composed of arginine and lysine, the fact that zeta potential changed from negative to positive confirmed the successful insertion of the TAT peptide. When the concentration of TAT reached about 1.2%, the particle size and zeta potential remained basically unchanged. This phenomenon could be attributed to the saturated insertion sites for HS-PEG-TAT on the surface of the nanoparticles.

### 3.2. pH-Responsive Charge Reversal of Shell/Core Composite Nanoparticles

**Figure 2:**
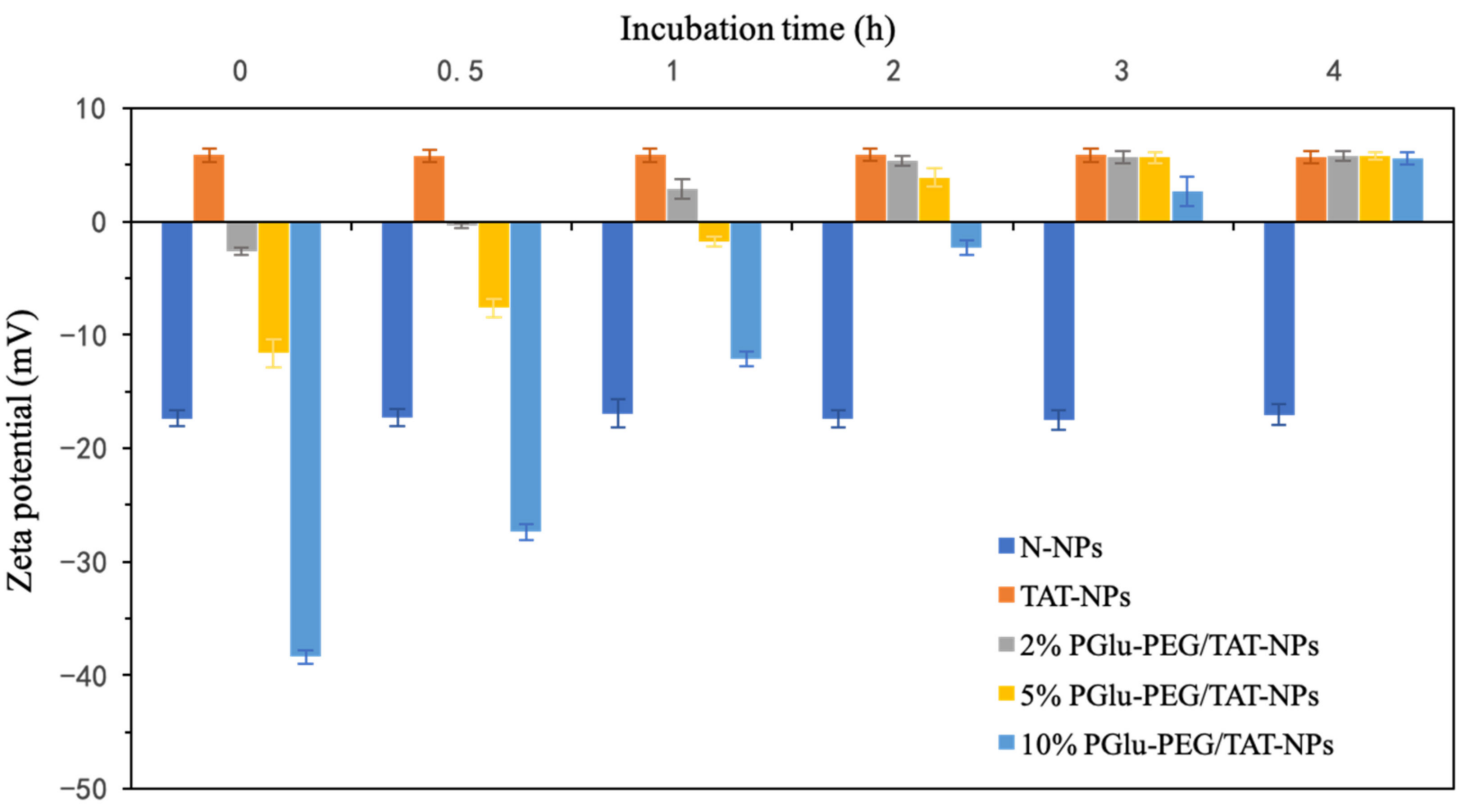
Changes in zeta potential of DSF-loaded N-NPs, TAT-NPs, PGlu-PEG/TAT-NPs with 2%, 5%, and 10% PGlu-PEG coating in pH 6.5 PBS during 4 h (*n* = 3). N-NPs, DSF-loaded naked nanoparticles; TAT-NPs, DSF-loaded cationic nanoparticles; PGlu-PEG/TAT-NPs, DSF-loaded shell/core composite nanoparticles.

The pH-responsive charge-reversal property of shell/core composite nanoparticles with different concentrations of PGlu-PEG (0, 2%, 5%, and 10%) was confirmed by monitoring the changes of zeta potential of PGlu-PEG/TAT-NPs incubated in PBS at pH 7.4 and pH 6.5. During a 24 h incubation at PBS pH 7.4, the zeta potential of each DSF-loaded formulation remained basically unchanged regardless of PEG coverage (Figure S4). However, in pH 6.5, we found an obvious charge-reversal process of PGlu-PEG/TAT-NPs, which was proportionally related to the percentage of PGlu-PEG coating. To be specific, compared with nanoparticles with 2% and 5% PGlu-PEG coating, the ones with 10% coating took more time to form a positively charged state. In general, nanoparticles with different PGlu-PEG coatings achieved charge reversal within 3 h. On the other hand, during incubation at PBS pH 6.5, it was observed that N-NPs and TAT-NPs still maintained stable negative and positive potentials, respectively, indicating the fact of their non-pH sensitivity (Figure 2). These results suggest that (i) PGlu-PEG/TAT-NPs, based on polyion complex of positively charged TAT and negatively charged PGlu-PEG, were equipped with excellent properties of pH-responsiveness, and that (ii) the pH-responsive efficiency of PGlu-PEG/TAT-NPs could be adjusted by the coating rate of PGlu-PEG in order to facilitate the subsequent charge-reversal process.

### 3.3. Plasma Stability Study

**Figure 3:**
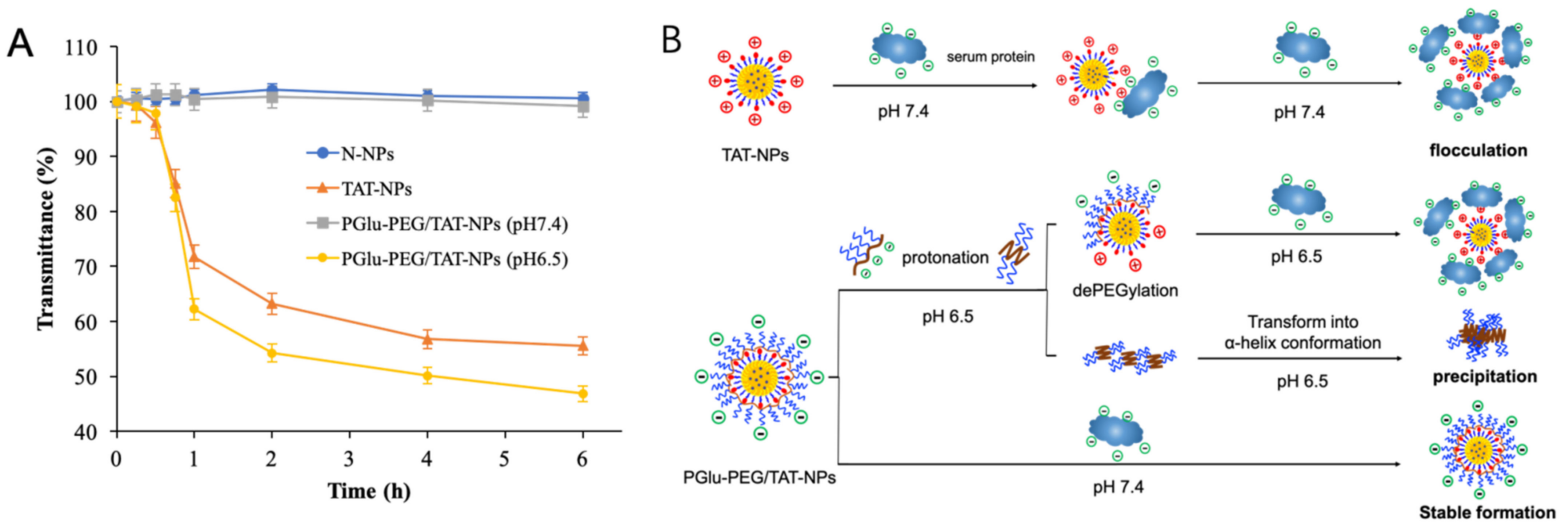
Stability of DSF-loaded nanoparticles in plasma. (**A**) Turbidity changes of N-NPs, TAT-NPs and PGlu-PEG/TAT-NPs pretreated with PBS containing 50% rat plasma of pH 7.4 and pH 6.5 (*n* = 3). (**B**) Supposed schematic illustration of the interaction between serum protein and DSF-loaded nanoparticles. N-NPs, DSF-loaded naked nanoparticles; TAT-NPs, DSF-loaded cationic nanoparticles; PGlu-PEG/TAT-NPs, DSF-loaded shell/core composite nanoparticles.

The positive charge of TAT renders it highly susceptible to interaction with the biological milieu, which might lead to destabilization and a rapid clearance of TAT-NPs from the reticuloendothelial system (RES) [30]. Consequently, the plasma stability of the TAT-modified delivery system becomes an essential requisite for further effective accumulation of NPs in tumor sites. The colloidal stability of DSF-loaded nanoparticles was firstly studied in rat plasma by monitoring the variations of transmittance, which were used to represent turbidity according to a previous study [31]. The initial transmittance was set as 100%, and the ones calculated at the time points that followed were set as a percentage of the initial value. As shown in Figure 3A, the transmittance of N-NPs and PGlu-PEG/TAT-NPs (pH 7.4) slightly changed within 6 h, indicating that both nanoparticles had good colloidal stability in plasma. However, under the condition of pH 6.5, the transmittance of PGlu-PEG/TAT-NPs markedly decreased to 46.9% after 6 h, which was similar to the situation of TAT-NPs (55.6%). According to the results, it is proposed that positive charged TAT-NPs could be attached to serum proteins to form flocculation, resulting in instability in systemic circulation (Figure 3B). On the other hand, based on the turbidity results of PGlu-PEG/TAT-NPs in different pH medium, we suggest that the PEG corona guaranteed a stable formation of PGlu-PEG/TAT-NPs against the presence of serum proteins. In addition, by means of abundant carboxylic groups, PGlu-PEG would be protonated and then lead to dePEGylation once the environmental pH value became 6.5. Simultaneously, the coil-like conformation of PGlu-PEG would be transformed into α-helix conformation with decreased solubility to form a precipitate, which would be manifested as a decrease in transmittance (Figure 3) [16]. These results indicate that PEG-coating is indispensable for TAT-NPs to increase their stability in circulation and that the fabrication with PEG might burst to be separated in the acidic conditions of the TME due to the protonation of PGlu-PEG.

**Figure 4:**
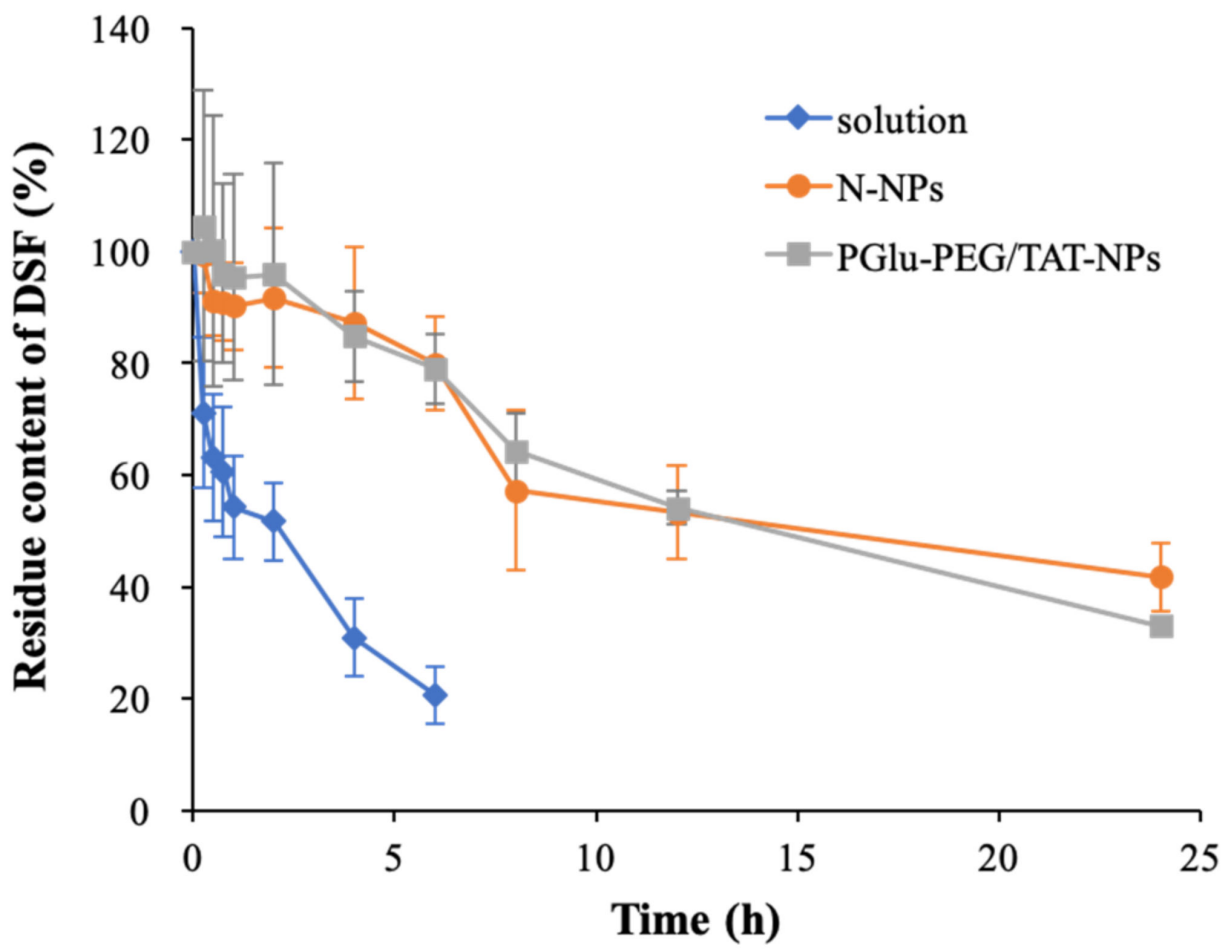
Degradation curves of DSF in solution as well as N-NPs and PGlu-PEG/TAT-NPs in rat plasma (*n* = 3). Solution, DSF dissolved in saline with a low content of Cremophor EL/ethanol; N-NPs, DSF-loaded naked nanoparticles; PGlu-PEG/TAT-NPs, DSF-loaded shell/core composite nanoparticles.

In addition to colloidal stability, the capability of nanoparticles to hold DSF is also a consideration in effective drug delivery. It is well known that free DSF is readily reduced to diethyldithiocarbamic acid and further metabolized in plasma, which limits its antitumor effect in clinical application [32]. As shown in Figure 4, nearly 80% of free DSF degraded within 6 h, among which ~50% was eliminated in the first 1 h; while the amount of degraded DSF was only about 20% in both PGlu-PEG/TAT-NPs and N-NPs. This suggested an improved stability of DSF depending on the encapsulation of nanoparticles. Moreover, the detectable amount of DSF still retained 30–40% of the initial amounts in these two nanoparticle delivery systems after 24 h. No statistically significant difference (*p* > 0.05) was observed between N-NPs and PGlu-PEG/TAT-NPs in the degradation content of DSF. However, no DSF was detected in the free DSF group (solution), indicating longer half-lives of DSF enveloped in nanoparticles than that of free DSF. Furthermore, the remaining concentration of DSF (c_t_, lnc_t_, and 1/c_t_) versus time were linearly regressed, and the results were fitted according to zero-order, first-order, and second-order kinetic processes. The results suggest that the degradation of DSF in plasma was in accordance with the first-order kinetic process. The degradation dynamics equation in the plasma can be expressed as ln(c_t_/c_0_) = −kt, where k is the degradation rate constant [33]. The half-lives of different DSF-loaded samples were calculated according to the equation t_1/2_ = 0.693/k (Table 2). Accordingly, we found that the half-lives of DSF were extended to almost 6-fold and 5-fold by encapsulating into N-NPs (18.6 ± 7.2 h) and PGlu-PEG/TAT-NPs (14.5 ± 4.7 h), respectively, compared with that of DSF solution (3.1 ± 1.3 h) in rat plasma, probably attributable to the protectively shell/core structure of nanoparticles. These results indicate that the inner lipid core showed a huge loading capability for DSF that could reduce its leakage and degradation in plasma, thereby promising to achieve longer circulating time than solution in vivo.

### 3.4. pH-Responsive Cellular Uptake Study

**Figure 5:**
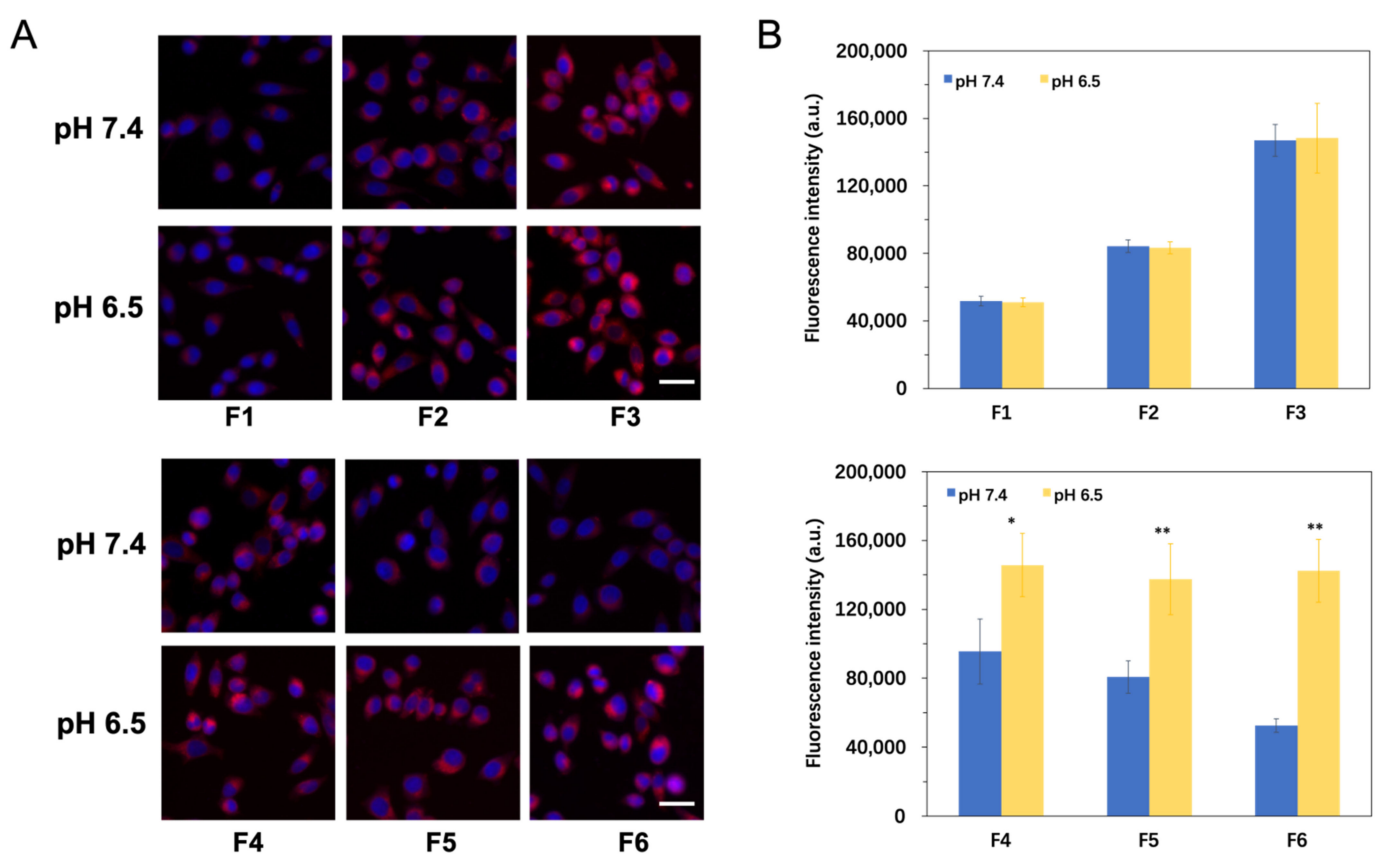
In vitro cellular uptake studies of Cy5.5-labeled nanoparticles with different concentrations of TAT and PGlu-PEG in Hep G2 cells for 4 h. (**A**) Representative images depicting cellular uptake of different formulations with various concentrations of TAT and PGlu-PEG coating (F1–F6) at pH 7.4 and pH 6.5; Blue: DAPI for nuclei, Red: Cy5.5. Scale bar represents 20 μm. (**B**) Quantitative analysis of the fluorescence signals of Cy5.5 at pH 7.4 and pH 6.5 (*n* = 3). * *p* < 0.05; ** *p* < 0.01. F1 represents N-NPs (without TAT or PGlu-PEG modifications); F2 and F3 represent TAT-NPs modified with different TAT concentrations (0.6% for F2 and 1.2% for F3) but without PGlu-PEG modification; F4, F5, and F6 represent PGlu-PEG/TAT-NPs thta were modified with the same TAT concentration (1.2%) but different PGlu-PEG concentrations (2% for F4, 5% for F5, and 10% for F6).

Cy5.5-loaded nanoparticles composed of different modifications were incubated with Hep G2 cells under two conditions (pH 7.4 and pH 6.5). The treated cells were photographed and analyzed by an ImageXpressMicro high content system (Figure 5A). After incubation in medium of pH 7.4, cells treated with TAT-NPs (F2 and F3) showed higher Cy5.5 fluorescence intensity compared with N-NPs (F1) due to increased cellular internalization via the electric attraction of poly cations and cell membrane (Figure 5B) [34]. What is more, we found that the fluorescence intensity gradually enhanced when the density of TAT increased from 0.6% (F2) to 1.2% (F3). As an aside, this confirmed the fact that the modification of TAT helped increase cell uptake. Meanwhile, cells treated with PGlu-PEG/TAT-NPs (F4, F5 and F6) exhibited lower fluorescence intensity compared with TAT-NPs (F3), owing to the steric hindrance of PEG chains that isolated an effective contact between TAT and Hep G2 cells [35]. The fluorescence intensity weakened as the percentage of PGlu-PEG increased from 2% (F4) to 10% (F6). It is also worth mentioning that when the percentage of PGlu-PEG reached 10% (F6), the Cy5.5 fluorescence intensity was close to that of the N-NPs (F1), indicating a complete shielding of TAT.

However, under the condition of pH 6.5, PGlu-PEG/TAT-NPs (F4, F5, and F6) showed enhanced intracellular fluorescent intensity, which was in sharp contrast to that in the condition of pH 7.4 (Figure 5C). Additionally, the variation of pH values from 7.4 to 6.5 did not induce significant change in the intracellular fluorescence intensity of either N-NPs (F1) or TAT-NPs (F2 and F3), indicating that the two did not have the property of responding to environmental pH. Once TAT-NPs were complexed with polyglutamic acids, they began to show sensitivity to the variation of environmental pH. By comparing the fluorescence intensity of nanoparticles with post-inserted TAT and PGlu-PEG coating (F3, F4, F5, and F6) in the medium of pH 6.5, we found that there was no significant difference in the uptake of Cy5.5 with regard to the PGlu-PEG coating density. This might result from a complete disassociation of the PGlu-PEG shell from the TAT-NPs core. These results indicate that i) the post-insertion of TAT could promote the internalization of nanoparticles in the tumor cells, and that ii) under the condition of pH 6.5, PGlu-PEG/TAT-NPs realized responsive uncoating and exposed TAT to facilitate the endocytosis of nanoparticles into tumor cells.

### 3.5. Cytotoxicity Study

**Figure 6:**
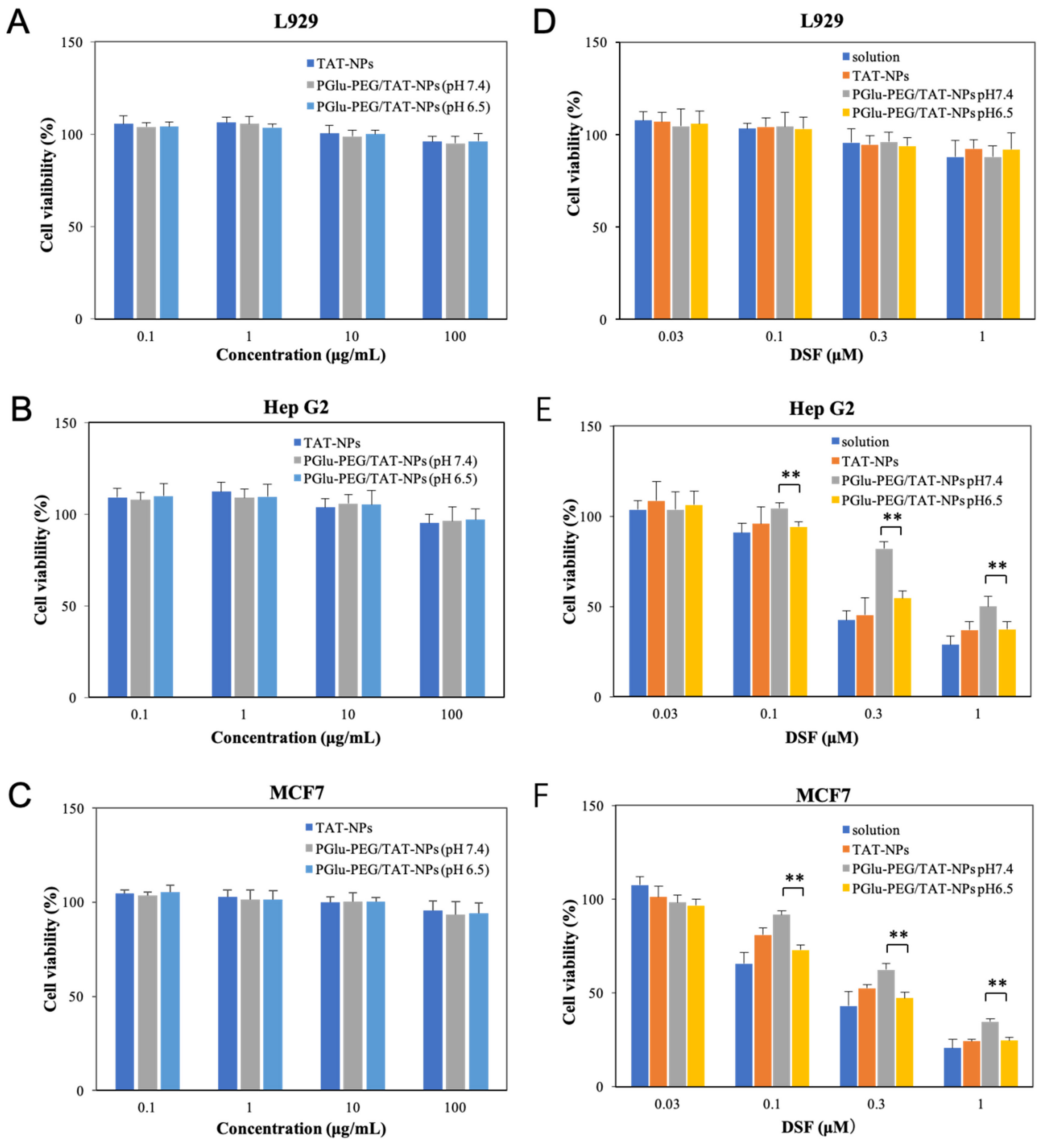
In vitro cytotoxicity of different preparations. Cell viability of L929 cells (**A**), Hep G2 cells (**B**), and MCF7 cells (**C**) after incubation with blank TAT-NPs and PGlu-PEG/TAT-NPs (pretreated with PBS of pH 7.4 or pH 6.5) for 24 h; cell viability of L929 cells (**D**), Hep G2 cells (**E**), and MCF7 cells (**F**) after incubation with DSF solution, DSF-loaded TAT-NPs, and DSF-loaded PGlu-PEG/TAT-NPs (pretreated with PBS of pH 7.4 or pH 6.5) for 24 h (*n* = 3). ** *p* < 0.01. Solution: DSF dissolved in saline with a low content of Cremophor EL/ethanol. TAT-NPs: cationic nanoparticles with TAT modification; PGlu-PEG/TAT-NPs: shell/core composite nanoparticles with TAT and PGlu-PEG/TAT-NPs modification.

The cytotoxicity of blank nanoparticles and DSF-loaded nanoparticles with different modifications of TAT and PGlu-PEG was studied on L929, Hep G2, and MCF7 cells by MTT assay. As shown in Figure 6A–C, the blank nanoparticles exhibited no cytotoxicity in cancer cell lines (MCF-7 and Hep G2) or normal cell line (L929) over various concentrations, ranging from 0.1 to 100 μg/mL after 24 h incubation, suggesting high biocompatibility and low toxicity of blank NPs. Relatively, DSF-loaded nanoparticles exhibited strong inhibition in the cell viability of Hep G2 and MCF7 cells, but little toxicity in L929 (Figure 6D–F), indicating the more selective toxicity of DSF towards cancer cells. Similar results were observed in previous research [36,37].

As shown in Figure 6E–F, DSF solution and TAT-NPs showed relatively high cytotoxicity due to the freely passive diffusion of small molecules across the cell membrane and increased internalization by means of cell-penetrating peptides, respectively. On the other hand, the cytotoxicity of PGlu-PEG/TAT-NPs showed low cytotoxicity at pH 7.4 due to the shielding effect of PGlu-PEG coating, which to some extent affected the uptake efficiency of nanoparticles in tumor cells. Meanwhile, increased cytotoxicity was detected at pH 6.5 since the PEG shell was detached from the nanoparticles, causing the exposed TAT to further enter the tumor cells. Finally, there was no significant difference of cytotoxicity between TAT-NPs and PGlu-PEG/TAT-NPs pretreated with PBS of pH6.5 (*p* > 0.05). The result of the cytotoxicity study is consistent with that of cellular uptake.

### 3.6. In Vivo Antitumor Study

**Figure 7:**
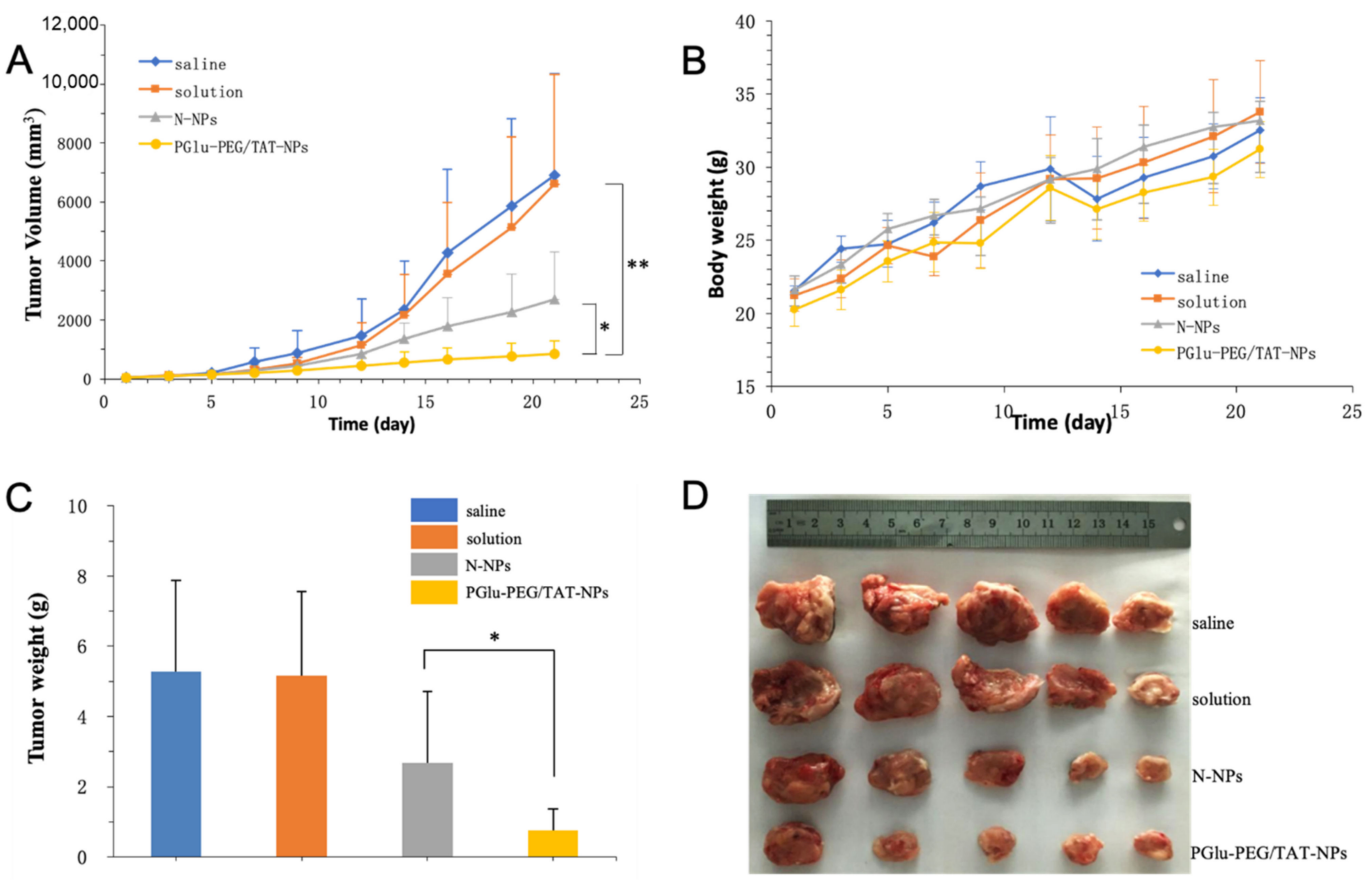
In vivo antitumor study of PGlu-PEG/TAT-NPs in H22 xenograft tumor. (**A**) Tumor growth curves following the treatment with saline, solution, N-NPs, and PGlu-PEG/TAT-NPs. (**B**) Mice body weight variations during treatment. (**C**) Weight of dissected tumors after treatment (*n* = 5). * *p* < 0.05; ** *p* < 0.01. (**D**) Picture of tumors after treatment. Solution: DSF dissolved in saline with a low content of Cremophor EL/ethanol. N-NPs: DSF-loaded naked nanoparticles. PGlu-PEG/TAT-NPs: shell/core composite nanoparticles.

The in vivo antitumor efficacy of DSF-loaded nanoparticles was studied in Kunming mice bearing H22 xenografts. Both DSF-loaded nanoparticles exhibited substantial growth inhibition of tumors in comparison with saline and solution groups (Figure 7A). It was observed that there was no drastic fluctuation in mice body weights (Figure 7B). PGlu-PEG/TAT-NPs showed an improved tumor inhibition rate, compared with DSF solution and N-NPs (PGlu-PEG/TAT-NPs: 80.68%; N-NPs: 43.22%; solution: 5.34%) (Figure 7A–D). It was worth noting that the DSF solution treatment group exhibited a similar tumor inhibition rate with the saline control group with almost no antitumor therapeutic effect. We suggest that this could be partially explained by the fact that DSF is unstable in the circulation and that it is easily degraded into diethyldithiocarbamic acid (DDC), which is further degraded into carbon disulfide and ethylamine [38]. The nanotechnology strategy based on a pH-responsive charge conversional shielding system equipped DSF with an excellent circulation stability, which is also an important prerequisite for DSF to play a role in the treatment of cancer. Additionally, such pH-triggered charge-reversal effectiveness endows PGlu-PEG/TAT-NPs with the TME targeting property that was ascribed to the low pH levels in tumor pathophysiology as compared with physiological pH levels exhibiting a stronger antitumor effect in comparison with solution and N-NPs (Figure 7C,D).

**Figure 8:**
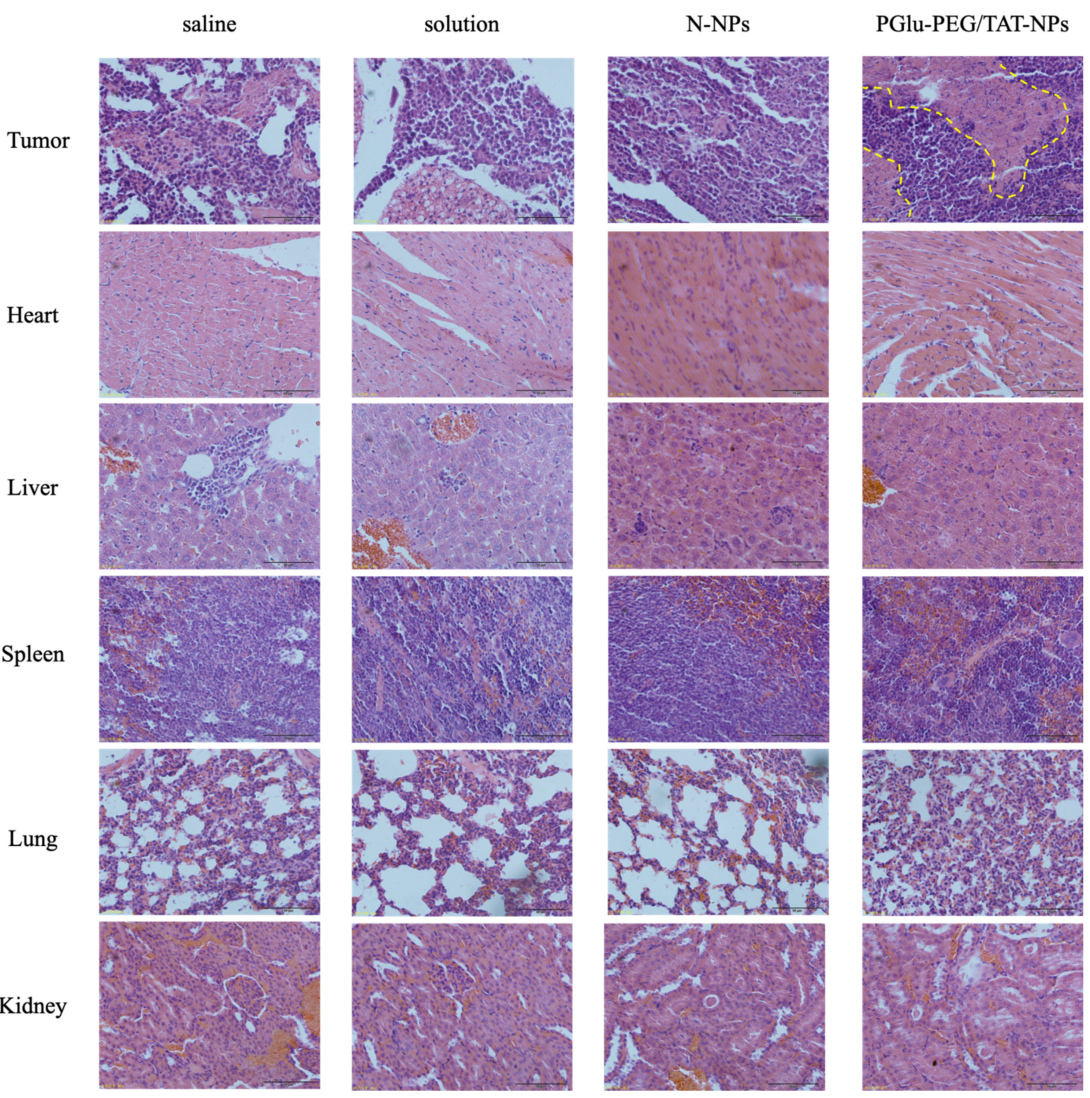
Light microscopy images of H&E staining tumor and major organs after the treatment of saline, solution, N-NPs, and PGlu-PEG/TAT-NPs. Scale bar represents 50 μm. Solution: DSF dissolved in saline with a low content of Cremophor EL/ethanol. N-NPs: DSF-loaded naked nanoparticles. PGlu-PEG/TAT-NPs: shell/core composite nanoparticles. Yellow dotted lines: necrotic and apoptotic cells region.

After H&E staining, it was observed that PGlu-PEG/TAT-NPs could induce more necrotic and apoptotic cells in a tumor over other groups (Figure 8). No significant pathological changes were observed in main organs (Figure 8), with little fluctuation in mice body weights (Figure 7B), indicating the low toxicity of DSF and safe biocompatibility of nanoparticles. This antitumor study suggested that PGlu-PEG/TAT-NPs could provide a better antitumor efficacy with low side effects compared with solution and N-NPs without the modification of PGlu-PEG and TAT peptide.

## 4. Conclusions

In this study, we reported the development and optimization of TME-responsive shell/core composite nanoparticles for enhanced stability and antitumor efficiency based on a pH-triggered charge-reversal mechanism. DSF-loaded nanoparticles were functionalized with post-insertion of TAT and polyion complex coating of PGlu-PEG. The optimized PGlu-PEG/TAT-NPs possessed a large loading efficiency of DSF with enhanced plasma stability as well as pH-responsive cytotoxicity and tumor cell uptake that finally resulted in a significant inhibition of tumor growth. Moreover, PGlu-PEG/TAT-NPs did not elicit a noticeably detrimental effect on major organs, suggesting its adequate biocompatibility. In view of this, nanoparticles equipped with pH-triggered charge-reversal properties are found to be promising carriers of chemotherapeutics with great potential in cancer therapy, benefiting from long-circulating stability and effective cell uptake via TAT insertion and fusogenicity.

## Figures and Tables

**Table 1:** Characterization of DSF-loaded nanoparticles with different modifications (*n* = 3).

Formulation	F1	F2	F3	F4	F5	F6
TAT ^a^ (%)	0	0.6	1.2	1.2	1.2	1.2
PGlu-PEG ^b^ (%)	0	0	0	2	5	10
Particle size (nm)	58.5 ± 1.2	63.3 ± 1.8	65.4 ± 2.7	68.3 ± 3.4	74.2 ± 2.1	87.4 ± 5.8
Zeta potential (mV)	−16.43 ± 1.22	3.03 ± 2.43	5.85 ± 2.28	−2.64 ± 2.16	−11.63 ± 3.48	−38.38 ± 2.15
DL (%)	5.16 ± 0.37	5.13 ± 0.44	5.26 ± 0.52	4.81 ± 0.45	4.93 ± 0.56	4.87 ± 0.24
EE (%)	94.7 ± 4.6	93.5 ± 5.7	93.7 ± 4.2	94.2 ± 3.9	94.6 ± 5.8	94.2 ± 4.0

^a^ TAT, AYGRKKRRQRRR; ^b^ PGlu-PEG, poly (glutamate acid)-graft-poly (ethylene glycol).

**Table 2:** Degradation kinetic equations and parameters of DSF in rat plasma.

Formulation	First-Order Kinetic Equation	R	K (h^−1^)	t_1/2_ (h)
Solution ^a^	ln(c_t_/c_0_) = −0.0979t + 1.89	0.970	0.225	3.07
N-NPs ^b^	ln(c_t_/c_0_) = −0.162t + 1.98	0.977	0.037	18.6
PGlu-PEG/TAT-NPs ^c^	ln(c_t_/c_0_) = −0.0208t + 2.00	0.993	0.048	14.5

^a^ Solution: DSF dissolved in saline with a low content of Cremophor EL/ethanol. ^b^ N-NPs: DSF-loaded naked nanoparticles; ^c^ PGlu-PEG/TAT-NPs: DSF-loaded shell/core composite nanoparticles.

## Data Availability

The data presented in this study are available on request from the corresponding author. The data are not publicly available due to privacy.

## Supplementary Materials

The following are available online at https://www.mdpi.com/article/10.3390/pharmaceutics13060895/s1, Figure S1. Mass spectrum of HS-PEG-TAT. Figure S2. Effect of post-insertion temperature and time on particle size (A) and zeta potential (B) of TAT-NPs. Figure S3. Particle size (A) and zeta potential (B) variation of TAT-NPs according to the concentration of TAT. Figure S4. Changes in zeta potential of DSF-loaded N-NPs, TAT-NPs, PGlu-PEG/TAT-NPs with 2%, 5% and 10% PGlu-PEG coating in pH 7.4 PBS during 24 h.

## Abbreviations

NDDS	nanoscale drug delivery systems
DSF	disulfiram
TME	tumor microenvironment
PGlu-PEG	poly (glutamate acid)-graft-poly (ethylene glycol)
HS-PEG-TAT	12-hydroxystearic-poly(ethylene glycol)-TAT
N-NPs	naked nanoparticles
TAT-NPs	TAT-inserted nanoparticles
PGlu-PEG/TAT-NPs	shell/core composite nanoparticles
TEM	transmission electron microscopy
PBS	phosphate buffer
TIR	tumor inhibition rate
H&E	hematoxylin and eosin
