# Supplementary material for: Tumor Microenvironment-Responsive Shell/Core Composite Nanoparticles for Enhanced Stability and Antitumor Efficiency Based on a pH-Triggered Charge-Reversal Mechanism
Source: Pharmaceutics. 2021 Jun 16;13(6):895. doi: 10.3390/pharmaceutics13060895 (PMC8235205; doi:10.3390/pharmaceutics13060895)
Supplement: Supplementary file 1 [file pharmaceutics-13-00895-s001.zip › pharmaceutics-1205379-supplementary.pdf]

pharmaceutics

Supplementary Materials: Tumor Microenvironment-Responsive Shell/Core Composite Nanoparticles for Enhanced Stability and Antitumor Efficiency Based on a pH-Triggered Charge-Reversal Mechanism

Qiuhua Luo , Wen Shi, Puxiu Wang, Yu Zhang, Jia Meng and Ling Zhang

Synthesis and characterization of HS-PEG-TAT

1. Synthesis

HS-PEG-TAT was synthesized by conjugating 12-hydroxystearic acid (HS), NHS-PEG16-maleimide and TAT peptide in a two-step reaction. The mixture of excess NHS-PEG16-maleimide and HS was stirring in anhydrous chloroform in the presence of triethylamine (TEA), followed by adding TAT peptide and kept stirring overnight at room temperature. The copolymer was purified by evaporating solvent and dialysis (MWCO 2000 Da). The final product was obtained by freeze-drying. The structure of the polymer was confirmed by MS analyses (Xevo TQ, Waters).

2. Characterization

The molecular weight of HS-PEG-TAT was confirmed by MS analysis. As shown in Figure S1, multiple hydrogenation peaks emerged due to positive charges of lysine and arginine in TAT. The molecular weight of polymer was 2760.6 by calculating through the peaks of [M+2H$^+$] and [M+3H$^+$], indicating the successful synthesis of HS-PEG-TAT.

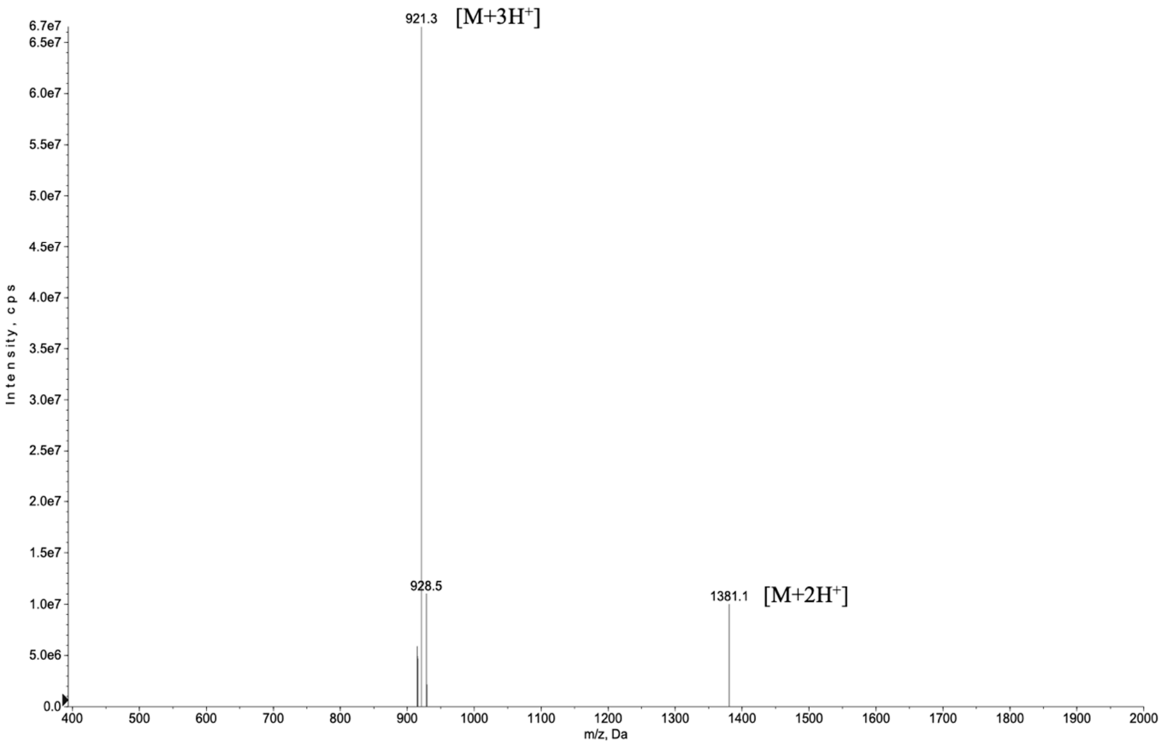

Figure S1. Mass spectrum of HS-PEG-TAT.

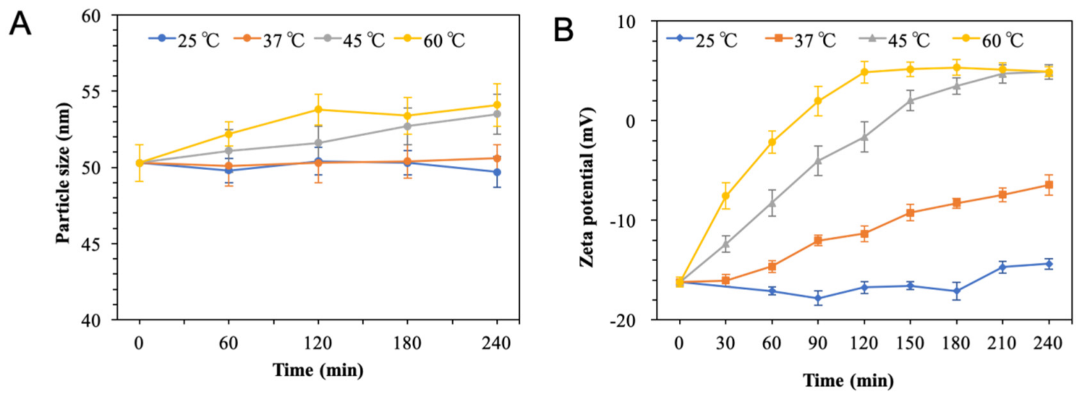

Figure S2. Effect of post-insertion temperature and time on particle size (A) and zeta potential (B) of TAT-NPs.

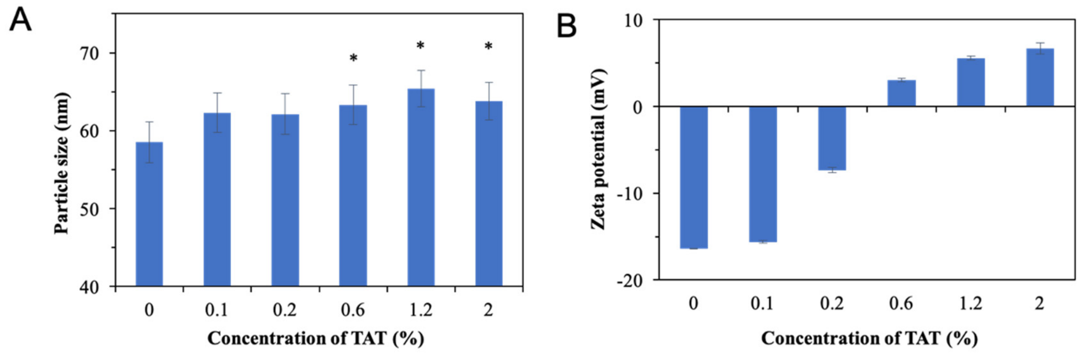

Figure S3. Particle size (A) and zeta potential (B) variation of TAT-NPs according to the concentration of TAT. *, $p<0.05$.

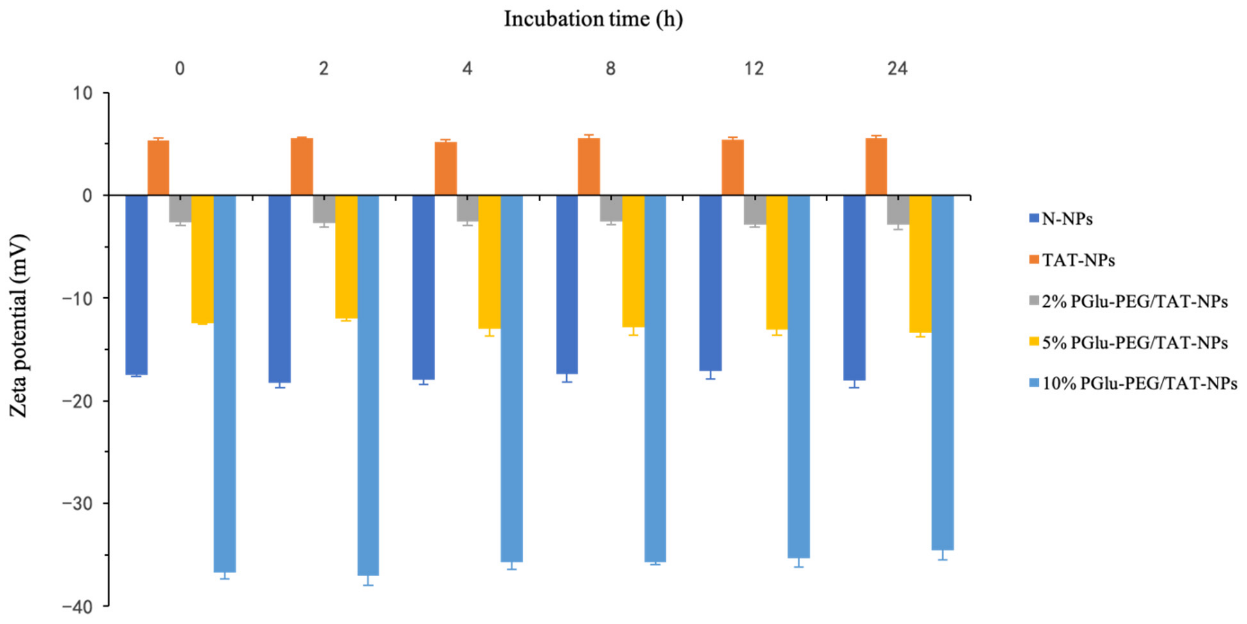

Figure S4. Changes in zeta potential of DSF-loaded N-NPs, TAT-NPs, PGlu-PEG/TAT-NPs with 2%, 5% and 10% PGlu-PEG coating in pH 7.4 PBS during 24 hours. N-NPs, DSF-loaded naked nanoparticles; TAT-NPs, DSF-loaded cationic nanoparticles; PGlu-PEG/TAT-NPs, DSF-loaded shell/core composite nanoparticles.
